# Therapeutic Targets and Molecular Mechanisms of Calycosin in the Treatment of Depression: Insights From Chronic Mild Stress Animal Models

**DOI:** 10.1111/cns.70353

**Published:** 2025-04-22

**Authors:** Guowei Gong, Yaqun Liu, Zhenxia Zhang, Yuzhong Zheng

**Affiliations:** ^1^ Department of Bioengineering Zunyi Medical University Zhuhai Guangdong China; ^2^ Guangdong Key Laboratory for Functional Substances in Medicinal Edible Resources and Healthcare Products, School of Life Sciences and Food Engineering Hanshan Normal University Chaozhou Guangdong China

**Keywords:** anti‐depressant, calycosin, CMS animal models, depression, underlying mechanisms

## Abstract

**Background:**

Depression is a complex psychiatric disorder with limited therapeutic options and various side effects. Calycosin, a bioactive compound derived from *Astragalus membranaceus*, possesses multiple pharmacological properties. This study aimed to investigate the antidepressant effects of calycosin in chronic mild stress (CMS) animal models of depression and to elucidate its underlying mechanisms.

**Methods:**

The antidepressant effects of calycosin were assessed in vivo using CMS animal models of depression, including the grooming frequency test, sucrose intake test, tail suspension test, and open field test. Neurogenic effects were evaluated by measuring the levels of BDNF, GDNF, and NGF in isolated hippocampus tissues. The hepatoprotective effects were assessed by measuring liver enzyme levels. The molecular mechanisms underlying calycosin's antidepressant effects were explored in vitro using PC12 cells.

**Results:**

Calycosin exhibited potent antidepressant‐like activities in CMS animal models of depression. Treatment with calycosin significantly alleviated depressive symptoms and improved neurogenic effects. Additionally, calycosin displayed hepatoprotective effects by modulating liver enzymes in vitro. The antidepressant effects of calycosin are mediated by the stimulation of the TrkB–MEK–Erk1/2–CREB signaling pathway.

**Conclusion:**

In conclusion, calycosin shows promise as a novel therapeutic agent for depression due to its potent antidepressant‐like activities and diverse pharmacological properties. Further studies are warranted to elucidate the exact molecular targets of calycosin and to assess its efficacy and safety in clinical settings.

## Graphical abstract

Calycosin modulates TrkB–MEK1/2–Erk1/2–CREB pathway for mitigating depression.

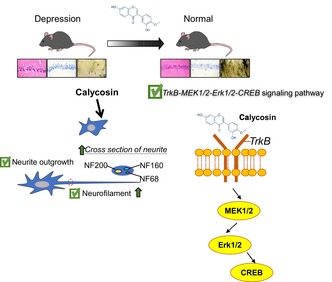

**Abbreviations**
CMS: chronic mild stress
DMEM: Dulbecco's modified Eagle medium
ECL: enhanced chemiluminescence
H&E: hematoxylin and eosin
HRP: horseradish peroxidase
PFA: paraformaldehyde
SNRIs: serotonin–norepinephrine reuptake inhibitors
SSRIs: selective serotonin reuptake inhibitors
TCM: traditional Chinese medicine

## 1 Introduction

Depression is a highly prevalent neuropsychiatric condition and debilitating mental health disorder that affects millions of individuals worldwide. It impacts over 300 million people across all age groups and stands as one of the primary contributors to the global disease burden [1]. This condition is characterized by persistent feelings of despair, a loss of hope, difficulties with decision‐making and concentration, fatigue, sleep disturbances, changes in appetite, a negative outlook, restlessness, and a lack of interest or pleasure in daily activities [2, 3]. The etiology of depression is complex, involving genetic, environmental, and neurological factors, making successful treatment challenging. Current pharmacological interventions, such as selective serotonin reuptake inhibitors (SSRIs, e.g., sertraline, fluoxetine, and paroxetine) and serotonin–norepinephrine reuptake inhibitors (SNRIs, e.g., desvenlafaxine, venlafaxine, and duloxetine), have limitations due to their side effects and their lack of effectiveness in a substantial proportion of patients [4, 5]. Less commonly prescribed antidepressant medications include serotonin 5‐HT2C receptor antagonists (such as olanzapine), alpha‐2 blockers (such as atipamezole), melatonin receptor agonists (such as ramelteon), and selective noradrenaline/dopamine reuptake inhibitors (such as nomifensine) [6].

Despite the availability of numerous types of antidepressant drugs, their overall effectiveness remains disappointingly low [7, 8]. Studies indicate that currently available antidepressants can only alleviate symptoms of moderate or severe depression in approximately 20% of adult patients [9]. Furthermore, antidepressant medication often requires a long‐term prescription to prevent relapse [10]. Moreover, more than half of individuals using antidepressants report experiencing side effects such as headaches, dry mouth, anxiety, dizziness, and weight gain [11, 12]. These side effects can often lead to discontinuation of antidepressant treatment in individuals with depression [13]. If left untreated, depression may lead to suicidal ideation or suicide attempts. Suicide ranks as the second most common cause of death among young adults worldwide, with approximately 800,000 cases reported every year [14]. This underscores the urgent need for novel therapeutic agents that can target multiple pathways implicated in depression with better efficacy and tolerability [15].

Herbal medicine or plant‐derived natural products can serve as a cost‐effective complementary and alternative treatment for depressive disorders, often with fewer side effects and comparable efficacy to conventional antidepressants [16]. Plant extracts are considered one of the most promising sources of new drugs and have shown promising results in the treatment of depression [17]. They have long been a valuable source of new drug candidates, particularly in the field of mental health, and are well‐tolerated by individuals with depression [18]. In recent years, there has been growing interest in identifying novel therapeutic agents from natural sources that have fewer side effects and can target multiple pathways implicated in depression [19, 20]. However, despite promising preclinical results, only a limited number of herbal medicines have successfully completed the clinical testing phase and received approval for use in clinical therapeutics.

Calycosin (7,3′‐dihydroxy‐4′‐methoxy isoflavone) is a bioactive phytoestrogen isoflavone found in various traditional Chinese medicinal foods such as *Astragalus membranaceus, Hedysarum polybotrys*, *Glycyrrhiza glabra* , and *Spatholobi caulis* [21, 22, 23]. Calycosin has garnered attention due to its multiple pharmacological properties, including hepatoprotective [24], neurogenic [25], cardioprotective [26], anti‐osteoporosis [23], pro‐angiogenesis [27], anti‐cancer [23, 28], and anti‐inflammatory effects [29]. Despite these known properties, there is currently a lack of research on the antidepressant effects of calycosin. The chronic mild stress (CMS) models, which involve pressure stimuli, are commonly used in research studies focused on treating depression with both synthetic agents and traditional Chinese medicine (TCM) [30]. In these studies, rodents are exposed to prolonged periods of chronic mild or unpredictable stress stimuli to induce behavior similar to that seen in depression [31]. The CMS model is now recognized as a crucial tool for assessing the efficacy of potential antidepressant compounds during the preclinical phase of research [32]. Hence, the aim of this present study is to investigate the therapeutic potential of calycosin in the treatment of depressive disorders and explore the underlying mechanisms responsible for its antidepressant‐like effects in CMS animal models.

## 2 Materials and Methods

### 2.1 Animal Study

C57BL/6 male and female mice weighing 24–26 g and aged 8–10 weeks were provided by Wuhan Shengyu Laboratory Animals Ltd. (Wuhan, China). The animals were kept in optimal conditions, which included a 12‐h light/dark cycle (lights on from 6 a.m. to 6 p.m., lights off from 6 p.m. to 6 a.m. the following day), a standard diet, unlimited water, a temperature of 22°C, and 50% humidity. The animal experimental protocol (No. ZMU21‐2202‐164) was approved by the Zunyi Medical University Animal Ethics Committee in accordance with the Principles of Laboratory Animal Care. The experimental environment was maintained free of light and noise to facilitate the development of the CMS‐induced depression model. Animals were randomly selected to form six groups, each containing six animals. The animals in the control group were given saline. The animals in the other groups underwent CMS procedures for a duration of 6 weeks.

The CMS procedures included the following: (1) 24 h of water deprivation; (2) 10 h of exposure to stroboscopic light; (3) 7 h in a 45° tilted cage; (4) 10 h in a noisy environment; (5) 12 h in a cage contaminated by 200 mL of water in 100 g of sawdust bedding; (6) 1 h in the presence of an empty bottle; (7) 6 min of forced swimming at 8°C; (8) 6 min of tail‐clipping; and (9) 21 h of food deprivation [19, 20]. These animals were then given various drugs intraperitoneally for an additional 2 weeks. As a positive control, imipramine (20 mg/kg) from Sigma–Aldrich (St. Louis, MO) was administered. For the experiments, three different concentrations of calycosin were used: 3 mg/kg (labeled ‐L), 10 mg/kg (labeled ‐M), and 30 mg/kg (labeled ‐H). Sigma–Aldrich was also the source of calycosin. The mice were anesthetized with general anesthesia using anesthetic agents (ketamine at 100 mg/kg and xylazine at 10 mg/kg) after conducting the behavioral tests.

The rationale behind the selection of doses and treatment durations was based on relevant literature, preliminary experiments, and the objectives of the study [33, 34, 35]. For the animal study, the doses of calycosin were chosen based on previous research indicating therapeutic benefits in animal models [29, 36, 37, 38]. The treatment duration was determined to allow sufficient time for observable effects on behavior and biochemical markers. In the cellular study, the concentration of calycosin was optimized using the MTT assay to identify the optimal dosage for promoting differentiation in PC12 cells. The preliminary data on the optimal dosage, as shown in Figure S1, guided the selection of treatment concentrations for the study.

### 2.2 Behavioral Assessments

#### 2.2.1 Grooming Frequency Test

In the study, the frequency of body grooming with the front paws was measured over a 5‐min period. Two observers, who were blinded to the animal groups, independently recorded the grooming frequency for each group.

#### 2.2.2 Sucrose Consumption Test

Sucrose preference tests were conducted at the end points of the experiment. On the first day, all animal groups were acclimated to two bottles containing a 1% sucrose solution (w/v). After 24 h, one of the bottles was replaced with tap water. The animals were then subjected to food and water restrictions for the remainder of the day. On the day of the test, the animals were placed in individual cages with access to two bottles: one containing water and the other containing a 1% sucrose solution (w/v). After 3 h, the amount of sucrose solution consumed was measured [19, 20]. The observers were blinded to the animal groups during the test.

#### 2.2.3 Tail Suspension Test

The tail suspension test was conducted in a controlled environment that was acoustically and visually isolated. Each animal was suspended by its tail, approximately 1 cm from the tip, using adhesive tape attached to a vertical bar. The animal's activity was recorded using a camera positioned in front of the tail suspension test box. The duration of immobility during the last 4 min of the 6‐min test period was calculated as an indicator of depressive‐like behavior [19, 20].

#### 2.2.4 Open Field Test

The day following the final medication treatment, open field tests were conducted using an apparatus made of white plastic. The open field area was divided into two regions: central and peripheral, each comprising 50% of the total space. The animals were transferred to the experimental room 1 h prior to the test for adaptation. Subsequently, they were placed in the open field chamber for 5 min under dim lighting conditions. The duration of time spent in the central area was automatically recorded during the test [19, 20].

### 2.3 Histological Staining

Following the behavioral assessment, the mice were euthanized and their brains were quickly removed. The brains were then fixed in a 4% paraformaldehyde (PFA) solution and later embedded in paraffin. The paraffin‐embedded brains were sectioned into 5‐μm‐thick slices [39]. Subsequently, Nissl staining and hematoxylin and eosin (H&E) staining were conducted on these brain slices using staining kits purchased from Abcam (Waltham, MA), following standard protocols and guidelines.

### 2.4 Golgi Staining

All animals in each group were anesthetized with isoflurane and then rapidly injected with a solution of PBS and 0.5% sodium nitrite, followed by 1 h of perfusion with 4% formaldehyde. Subsequently, the brains were treated with mordant dye, comprising a mixture of 5% chloral hydrate, 5% potassium dichromate, and 4% formaldehyde, for a duration of 3 h. Following this, the brains were immersed in the mordant dye for 4 days in darkness, then transferred to a 1.5% silver nitrate solution that was changed daily, over a total of 3 days. Finally, the tissues were sectioned into 50‐μm sections, fixed, and imaged [40].

### 2.5 Fluorescent Staining

The Olympus Fluoview FV1000 laser scanning confocal microscope, manufactured by Olympus America (Melville, NY) and mounted on an inverted Olympus microscope with a 40× objective, was used for the study. The tissue sections, sliced into 5‐μm thickness, were incubated with anti‐Syp and/or anti‐PSD95 antibodies at a 1:200 dilution (CST, Danvers, MA) for 24 h. In the in vitro experiment, the cells were fixed with 4% PFA for 10 min, followed by three washes with PBS. Immunostaining was carried out using the following primary antibodies: anti‐TrkB, anti‐MEK1/2 (both diluted 1:500), anti‐Erk1/2, and anti‐CREB (all diluted 1:500) (CST). The primary antibody was diluted in cold PBS containing 2.5% fetal bovine serum and 0.1% Triton X‐100. The secondary antibody used was a FITC‐labeled anti‐rabbit antibody (diluted 1:1000; Jackson Laboratories, West Grove, Pennsylvania) [19, 20].

### 2.6 SDS‐PAGE

The samples were homogenized using a high‐salt lysis buffer containing 1 M NaCl, 10 mM HEPES (pH 7.5), 1 mM EDTA, and 0.5% Triton X‐100. After centrifugation at 4°C for 10 min at 16,100 rpm, the supernatant was collected. Equivalent amounts of total protein were mixed with 2X lysis buffer (containing 0.125 M HCl, pH 6.8, 4% SDS, 20% glycerol, 2% 2‐mercaptoethanol, and 0.02% bromophenol blue) and subjected to SDS‐PAGE analysis. The membranes were then incubated overnight at 4°C with primary antibodies, including anti‐Syp, anti‐PSD95, anti‐TrkB, anti‐MEK1/2, anti‐Erk1/2, and anti‐CREB, all diluted at 1:1000 (CST), and anti‐GAPDH diluted at 1:10,000 (CST). After washing, the membranes were incubated for 3 h at room temperature with horseradish peroxidase (HRP)‐conjugated anti‐rabbit secondary antibodies diluted at 1:5000. Finally, the immune complexes were detected using the enhanced chemiluminescence (ECL) technique [19, 20].

### 2.7 Biochemistry Assay

The levels of 5‐HT, DA, NE, BDNF, GDNF, NGF, AST, ALT, and ALP were determined using a specific colorimetric testing kit purchased from Abcam, in accordance with the manufacturer's recommended guidelines [19, 20].

### 2.8 IHC Staining

Following the preparation of 5‐μm sections from each experimental group, primary antibodies against TrkB, MEK1/2, Erk1/2, and CREB were diluted to a 1:200 ratio and used for immunostaining. The sections were incubated with the primary antibodies overnight at a cold temperature. Subsequently, secondary antibodies were applied at a 1:500 dilution and allowed to incubate for 3 h. The stained sections were examined using a Nikon light microscope (Hokkaido, Japan), and images were captured [19, 20].

### 2.9 Culture of Cells

PC12 cells, purchased from the American Type Culture Collection, were cultured in DMEM (Dulbecco's Modified Eagle Medium) and incubated in a humidified incubator at 37°C with 5% CO_2_ [41].

### 2.10 Identification of Differentiated Cells

PC12 cells were cultured in DMEM supplemented with 1% FBS and 1% HS. Subsequently, the cells were treated with varying concentrations of calycosin or BDNF for a duration of 48 h. Images of the cells were captured using phase‐contrast light microscopy. Cells were classified as differentiated if their neurites extended beyond the diameter of the cell body.

### 2.11 Luciferase Assay

Cells were transfected with two promoter constructs, pNF‐68‐Luc and pNF‐160‐Luc, in addition to the target plasmid using Lipofectamine 3000 (CST), following the manufacturer's instructions. After 48 h, the cells were treated with varying drug doses in 24‐well plates containing cultured cells seeded at 6 × 10^4^ cells/mL. The medium was aspirated, and the cultures were rinsed twice with PBS. Subsequently, the cells were lysed at 4°C in a solution containing 0.2% Triton X‐100, 1 mM DTT, and 100 mM potassium phosphate buffer (pH 7.8). The supernatant was collected and centrifuged for 10 min at 13,200 rpm, followed by the performance of a luciferase assay.

### 2.12 Statistical Analysis

Protein concentrations were determined using Bradford's assay (Hercules, CA). All statistical analyses were performed using SPSS version 25.0. Continuous variables are presented as mean ± SD. Based on the data distribution, unpaired or paired Student's *t*‐tests, Wilcoxon rank‐sum tests, one‐way ANOVA, and normality tests were used to analyze differences between groups. A two‐tailed *p*‐value of < 0.05 was considered statistically significant.

## 3 Results

### 3.1 The In Vivo Anti‐Depressive Effects of Calycosin on the CMS Model

**FIGURE 1:**
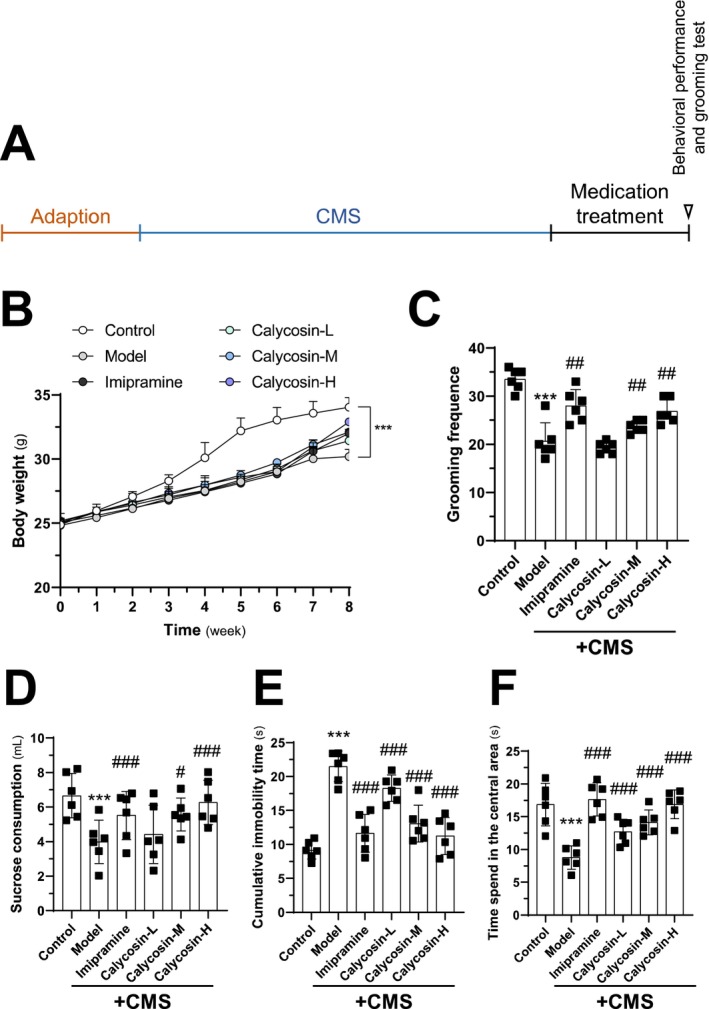
The in vivo behavior tests in CMS‐induced depressed C57BL/6 mice are affected by calycosin. (A) Diagram showing the process and design of the experiment. (B) Throughout the experiment, weekly body weight measurements of the animals were taken. The following tests were examined: (C) Mice grooming frequency; (D) sucrose consumption test; (E) tail suspension assay; and (F) open field test. There are six groups and six animals in each group. The calycosin‐L group (3 mg/kg), calycosin‐M group (10 mg/kg), and calycosin‐H group (30 mg/kg) were employed, with imipramine (20 mg/kg) serving as the positive control. The results are presented as mean ± SD, with *n* = 6. The control group was compared with ***p < 0.001, while the CMS model group was compared with ^#^ *p* < 0.05, ^##^ *p* < 0.01, and ^###^ *p* < 0.001.

Figure 1A illustrates the experimental design of the CMS animal study. Both the CMS model group and the control group received saline administration. For the final 2 weeks, mice were orally administered either calycosin or imipramine (20 mg/kg). The CMS animals were divided into three groups receiving different dosages of calycosin: 3 mg/kg (‐L), 10 mg/kg (‐M), and 30 mg/kg (‐H). Body weight measurements were taken once a week throughout the study to evaluate the effects of calycosin treatment. The body weights of all mice gradually increased over the course of the study (Figure 1B). Notably, the body weights of the control group differed significantly from those of the CMS model group (Figure 1B). In contrast to the CMS model group, the body weight increased after 2 weeks of calycosin intervention (Figure 1B).

To assess the antidepressant effects of calycosin, behavioral tests including grooming frequency, sucrose preference, tail suspension, and open field were conducted (Figure 1C–F). Grooming frequency was significantly lower in the CMS model group compared to the control group (Figure 1C). Treatment with calycosin resulted in increased grooming frequency in CMS mice, and the effect was dose‐dependent (Figure 1C). Sucrose consumption was significantly decreased in the CMS model group compared to the control group in vivo (Figure 1D). However, calycosin and/or imipramine treatment increased sucrose consumption in contrast to the CMS‐induced model groups (Figure 1D). In the tail suspension test, CMS‐induced depression in mice led to increased cumulative immobility time, while calycosin treatment significantly reduced immobility time in a dose‐dependent manner (Figure 1E). Finally, the CMS model animals exhibited decreased movement in the open‐field test compared to the control group. The time spent was reversed when calycosin was administered (Figure 1F).

### 3.2 The In Vivo Histological Analysis of the Effects of Calycosin on the CMS Model Hippocampal CA1 Region

**FIGURE 2:**
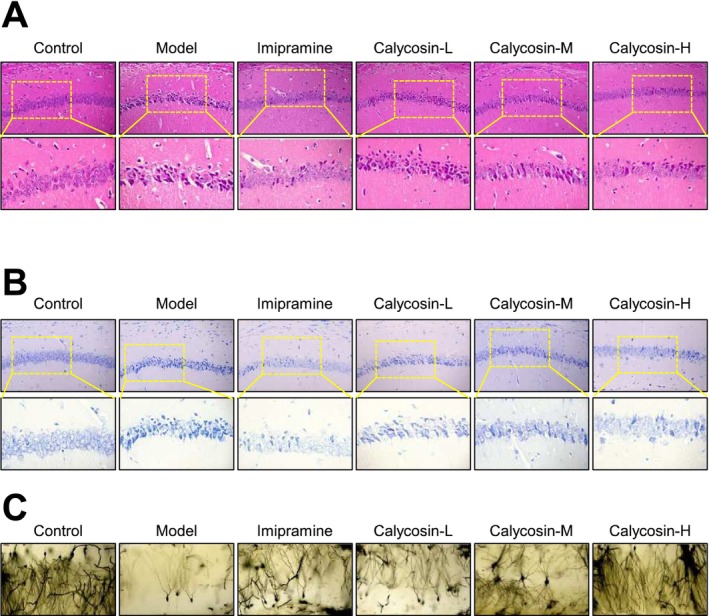
The in vivo impact of calycosin on the morphological alterations in the hippocampal CA1 region. (A) H&E staining of the various medication groups' hippocampal CA1 regions. (B) Nissl staining of the various medication groups' hippocampal CA1 regions. (C) By using Golgi staining, the dendritic morphology of neurons in the CA1 region was examined.

The in vivo histochemical experiment was conducted to analyze histological changes in animals after 2 weeks of calycosin treatment (Figure 2). First, the hippocampal CA1 region was stained with H&E to examine its morphological changes (Figure 2A). In the control group, nerve cells were well‐organized and roundly arranged, with intact cell structure, and an obvious cell membrane and nucleus. No visible swelling or necrosis was observed. In contrast, the nerve cells in the CMS model group exhibited disordered arrangement, irregular size, and shape. The number of nerve cells was significantly reduced in vivo, and the cell structure appeared blurred (Figure 2A). However, after 2 weeks of oral administration of 10 and/or 30 mg/kg calycosin, the nerve cells showed high integrity, and no obvious necrosis was observed (Figure 2A). Subsequently, Nissl staining was used to investigate the impact of calycosin on CA1 survival (Figure 2B). The CMS model group displayed a significant decrease in the number of positive neuron cells in the CA1 region compared to the control group. However, treatment with calycosin resulted in a dose‐dependent, significant increase in the number of positive neuron cells in the CA1 region compared to the CMS model group (Figure 2B). Finally, Golgi staining was performed to assess changes in spine density in the CA1 region and evaluate the impact of calycosin on neuronal synaptic plasticity. The findings revealed that the neurons in the model group had lower dendritic complexity compared to the control groups, as indicated by shorter dendritic length and fewer dendritic branches (Figure 2C). Mice with CMS that received imipramine or calycosin treatment exhibited longer and more dendrites overall (Figure 2C).

### 3.3 The In Vivo Analysis of Synaptic Plasticity Regulators and Translational Levels in Hippocampus CA1 Region in Response to Calycosin Treatment

**FIGURE 3:**
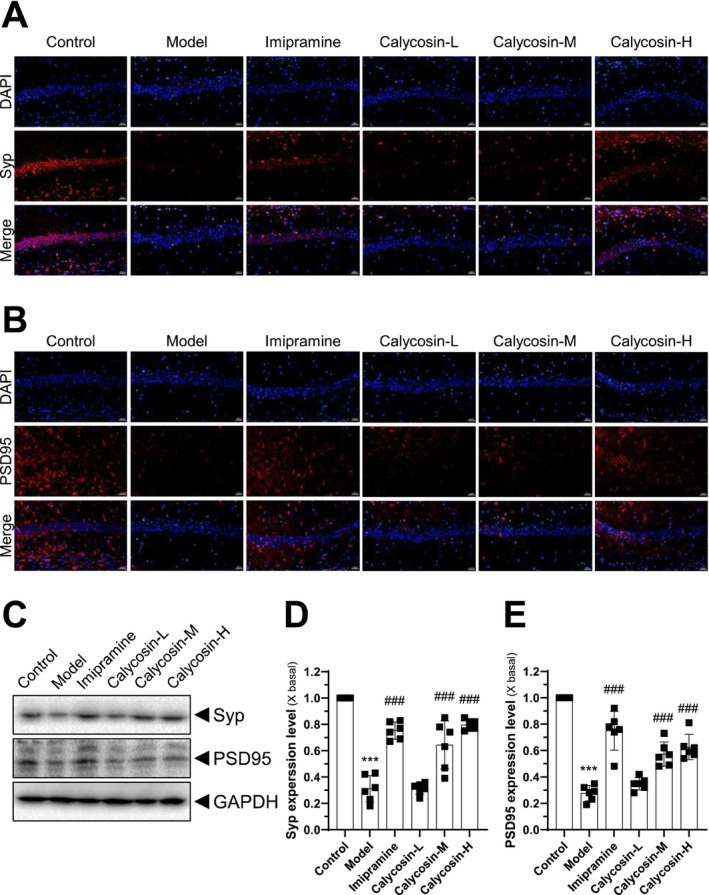
Calycosin increases the plasticity of synapses in the hippocampus CA1 region. The hippocampus CA1 region's immunofluorescence images of Syp (A) and PSD95 (B) after various drug treatments are depicted. Target genes were displayed in red, and the nuclei, which had been stained with DAPI, were displayed in blue. (C–E) Here is a display of the Syp and PSD western blotting analyses. GAPDH served as an internal loading control. Imipramine (20 mg/kg) was used as the positive control, and the calycosin‐L group (3 mg/kg), calycosin‐M group (10 mg/kg), and calycosin‐H group (30 mg/kg) were used. The results are presented as mean ± SD, with *n* = 6. The control group was compared with ***p < 0.001, while the CMS model group was compared with ^###^ *p* < 0.001.

To determine the expression levels of crucial synaptic plasticity regulators, PSD‐95 and SYP, we conducted immunoblotting using the methods described in Figure 3. The results revealed that the CMS model group exhibited lower levels of SYP and PSD‐95 in the hippocampal CA1 region compared to the control group, as indicated by the fluorescence intensities (Figure 3A,B). Conversely, treatment with imipramine and/or calycosin significantly increased the levels of SYP and PSD‐95 compared to the CMS model group in vivo (Figure 3A,B). Additionally, western blotting was used to assess the translational levels of the target genes (Figure 3C). The preliminary results indicated that PSD‐95 and SYP were dysfunctional in the model group (Figure 3C–E). However, the levels in the calycosin treatment groups were higher than those in the CMS model group in the CA1 region (Figure 3D,E). These findings suggest that calycosin has the potential to stimulate synapse formation in conditions resembling depression.

### 3.4 The In Vivo Analysis of Neurotransmitter Levels and Neurotrophic Factors in the Homogenized Hippocampus Following Calycosin Treatment

**FIGURE 4:**
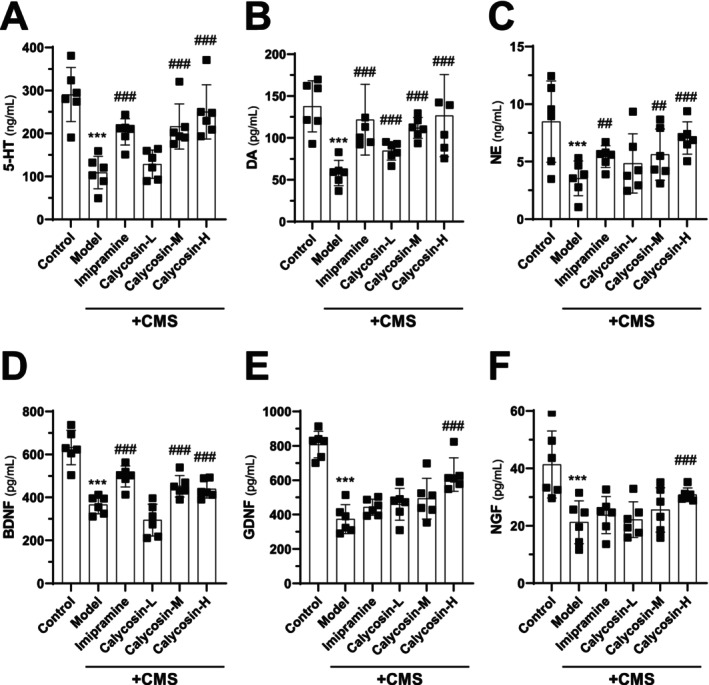
Calycosin increases the synthesis of NGF, GDNF, and BDNF as well as neurotransmitters in the hippocampal tissue. (A–C) The expression levels of 5‐HT, DA, and NE in the homogenized hippocampal tissue in different groups. (D–F) The homogenized hippocampal tissue's BDNF, GDNF, and NGF levels using the specific test kit. The calycosin‐L group (3 mg/kg), calycosin‐M group (10 mg/kg), and calycosin‐H group (30 mg/kg) were employed, with imipramine (20 mg/kg) serving as the positive control. The results are presented as mean ± SD, with *n* = 6. The control group was compared with ***p < 0.001, while the CMS model group was compared with ^##^ *p* < 0.01 and ^###^ *p* < 0.001.

Furthermore, the levels of neurotransmitters in the homogenized hippocampus tissue of mice in different treatment groups were measured using specific neurotransmitter testing kits (Figure 4A–C). The in vivo results showed that calycosin treatment significantly restored the production of neurotransmitters in mice that underwent the CMS procedure. In contrast, the levels of 5‐HT, DA, and NE in the hippocampus were significantly decreased in the model group compared to the control group (Figure 4A–C). Imipramine was used as a positive control, and after 2 weeks of treatment, the production of neurotransmitters was significantly increased compared to the model group (Figure 4A–C). The onset of depression has been associated with structural changes in the hippocampus and a decrease in BDNF, GDNF, and NGF [42, 43]. Therefore, the amounts of BDNF, GDNF, and NGF in the isolated hippocampal tissue of each animal were measured using an ELISA testing kit (Figure 4D–F). The results showed that the homogenized hippocampal expression levels of BDNF, GDNF, and NGF were significantly lower in the CMS group compared to the control group (Figure 4D–F). Animals treated with imipramine for 14 days exhibited higher levels of BDNF, GDNF, and NGF compared to the CMS model group. Similarly, calycosin treatment showed a trend of increasing BDNF, GDNF, and NGF levels after 2 weeks of intervention compared to the model group. Notably, calycosin at a higher concentration (30 mg/kg) demonstrated a robust activation in promoting the formation of BDNF, GDNF, and NGF compared to the lower concentration used (Figure 4D–F).

### 3.5 Investigation of the TrkB–MEK1/2–Erk1/2–CREB Signaling Pathway and Its Role in the Antidepressant Effect of Calycosin in Hippocampus

**FIGURE 5:**
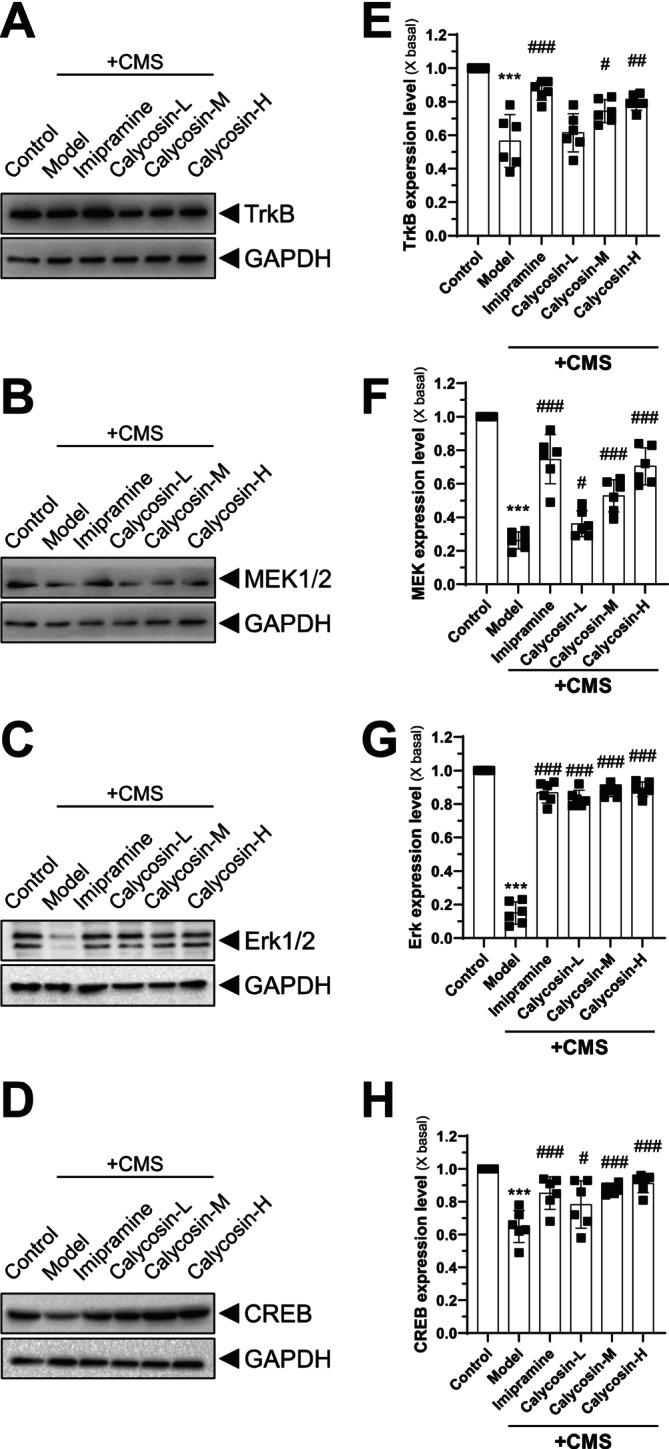
Calycosin increases the translational levels of the target genes in the hippocampal tissue. The animals were given varying doses of calycosin (3, 10 and 30 mg/kg), and after the animals were sacrificed, the homogenized hippocampal tissues were taken from each group. TrkB (A), MEK1/2 (B), Erk1/2 (C), and CREB (D) were identified using specific antibodies. GAPDH was used for loading control. The blot was quantified using a densitometer (E–H). The results are presented as mean ± SD, with *n* = 6. The control group was compared with ***p < 0.001, while the CMS model group was compared with ^#^ *p* < 0.05 and ^###^ *p* < 0.001.

**FIGURE 6:**
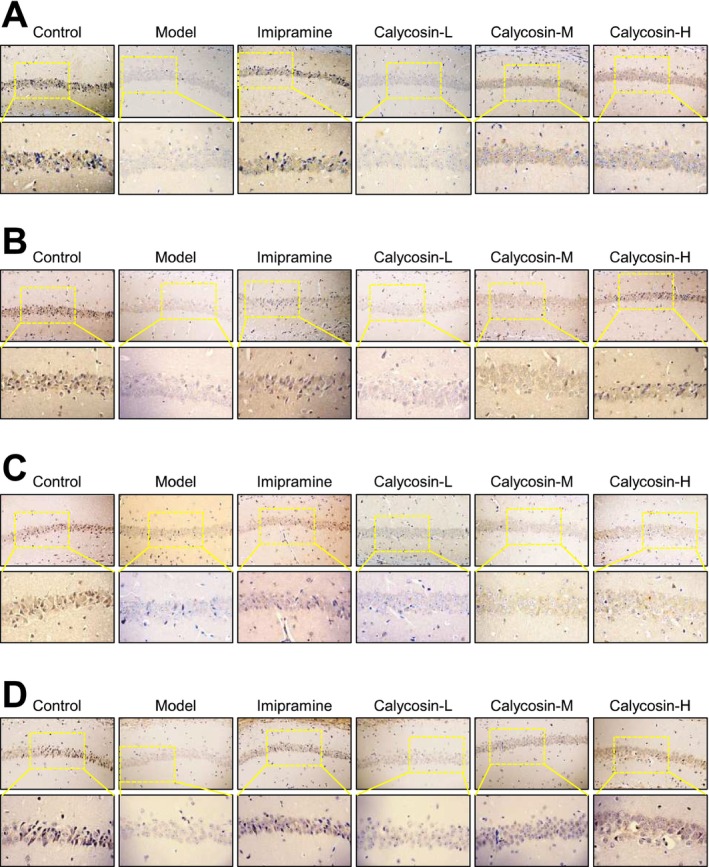
Using immunohistochemistry staining, calycosin increases the levels of TrkB‐MEK1/2‐Erk1/2‐CREB expression in the hippocampal CA1 region. Following sacrificing the animals and collecting hippocampal tissues from different groups, the hippocampal CA1 region was sliced into 5‐μm‐thick sections. Using immunohistochemistry, the expressions of TrkB (A), MEK1/2 (B), Erk1/2 (C), and CREB (D) were explored in the CA1 region.

An increasing body of evidence suggests that the TrkB–MEK1/2–Erk1/2–CREB signaling pathway plays a crucial role in the development of depression and synaptic plasticity in the hippocampus [44]. To elucidate the involvement of this pathway in the antidepressant effect of calycosin, we employed western blotting and IHC techniques to assess the protein levels of TrkB, MEK1/2, Erk1/2, and CREB in the hippocampus (Figures 5 and 6). Comparing the CMS model group to the control group, we observed a significant downregulation of TrkB (Figure 5A), MEK1/2 (Figure 5B), Erk1/2 (Figure 5C), and CREB (Figure 5D) protein expression in the homogenized hippocampal tissue, indicative of impaired signaling within the pathway (Figure 5). However, calycosin treatment dose‐dependently restored the translational levels of these target genes as reflected by the in vivo data, suggesting its potential to enhance synaptic plasticity in conditions resembling depression.

IHC further revealed a significant reduction in TrkB‐positive cells in the hippocampus CA1 region of the model group compared to the control group (Figure 6A). Notably, both the imipramine treatment group and the high‐dose calycosin group exhibited a significant increase in TrkB‐positive cells compared to the model group (Figure 6A). Similarly, the expression levels of MEK1/2‐positive cells (Figure 6B), Erk1/2‐positive cells (Figure 6C), and CREB‐positive cells (Figure 6D) in the CA1 region were significantly higher in the calycosin treatment groups, showing a dose‐dependent response. These findings highlight the potential of calycosin to activate the TrkB–MEK1/2–Erk1/2–CREB signaling pathway, promoting synaptic plasticity, and suggesting its role in the antidepressant effects of calycosin.

### 3.6 Bio‐Safety of Calycosin in CMS‐Exposed Mice

**FIGURE 7:**
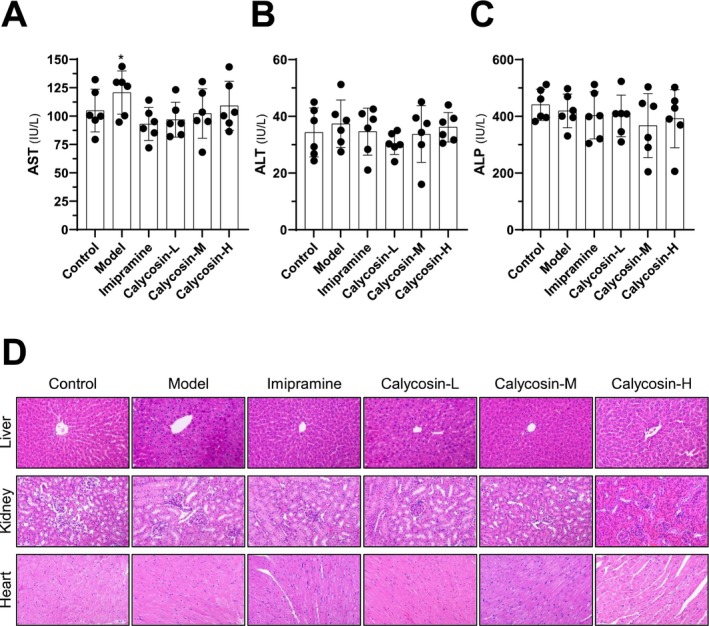
The in vivo preliminary biosafety determinations. Detection of AST (A), ALT (B), and ALP (C) in animal serum using a particular testing kit. (D) HE staining of the liver, kidney, and heart in mice treated with control, CMS model, imipramine, and calycosin (3, 10, and 30 mg/kg). The results are presented as mean ± SD, with *n* = 6. The control group was compared with *p < 0.05.

To investigate the potential hepatoprotective effects of calycosin in mice exposed to CMS, we measured the levels of serum liver enzymes including ALP, AST, and ALT (Figure 7A–C). The results revealed that the CMS model group exhibited significantly higher levels of serum ALT compared to the control group, indicating the presence of hepatic dysfunction associated with depression‐like syndromes (Figure 7A). However, treatment with calycosin showed a protective effect on hepatic tissues, as evidenced by the lower levels of serum liver enzymes in the calycosin‐treated groups (Figure 7A–C).

To further evaluate the histological changes in the liver, histology specimens were collected and examined under a microscope. Interestingly, no obvious morphological alterations or tissue abnormalities were observed in any of the groups (Figure 7D). Additionally, there were no detectable signs of renal tubular or glomerular abnormalities, hepatic inflammation, or heart fibrosis (Figure 7D). These findings suggest that calycosin may possess hepatoprotective properties, potentially mitigating the detrimental effects of depression‐like syndromes on liver function.

### 3.7 Investigation of Calycosin's Effect on Neuronal Differentiation in PC12 Cells as In Vitro Model

**FIGURE 8:**
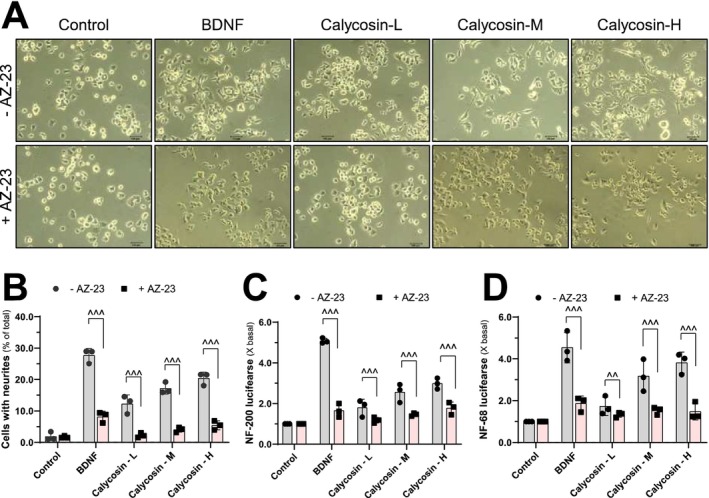
Calycosin promotes neuronal differentiation in vitro using PC12 cells. (A) Cultures were pre‐treated with medium (control) or AZ‐23 (5 nM) for 3 h before adding BDNF (20 ng/mL) or calycosin (1, 3, 10 μM) for 48 h. Representative images were displayed here. (B) The percentage of differentiated cells was determined as described in the Methods section. Following pNF68‐Luc (C) and pNF200‐Luc (D) transfection, the cells received a different medication. After 48 h of incubation, the luciferase activity of each sample was measured. The results are presented as mean ± SD, with *n* = 3. Statistically significant changes were classified as more significant ^^^^ *p* < 0.01, and highly significant ^^^^^ *p* < 0.001.

To explore the potential impact of calycosin on neuronal differentiation, we conducted in vitro experiments using PC12 cells. Neurite length was measured as an indicator of morphologically induced neuronal differentiation in response to calycosin treatment (Figure 8). It is well‐established that the three subunits of mammalian neurofilaments, NF68, NF160, and NF200, play a crucial role in the structural domain of neurites [45, 46]. In our study, BDNF treatment induced neurite outgrowth in cultured PC12 cells (Figure 8A). Interestingly, pre‐incubation of the BDNF‐treated cells with AZ‐23, a specific inhibitor of TrkB tyrosine phosphorylation, did not affect neurite outgrowth in vitro (Figure 8A,B). Similarly, calycosin treatment dose‐dependently promoted neurite outgrowth in PC12 cells. However, when PC12 cells were pretreated with AZ‐23, the calycosin‐induced neuronal differentiation was abolished (Figure 8A,B). To further investigate the biological role of calycosin in neuronal differentiation, we transfected the promoters of genes encoding neurofilaments (NF68 and NF200) linked to a luciferase reporter gene into PC12 cells (Figure 8C,D). Bioluminescence was used to assess the transcriptional activity of NF68 and NF200 in response to calycosin treatment. After 2 days of calycosin application, the in vitro transcriptional levels of pNF68‐Luc and pNF200‐Luc were induced in a dose‐dependent manner (Figure 8C,D). However, when PC12 cells were incubated with AZ‐23, the transcriptional levels of pNF68‐Luc and pNF200‐Luc triggered by calycosin were significantly lower compared to the untreated group (Figure 8C,D). These results suggest that calycosin‐induced neuronal differentiation is mediated through TrkB signaling. Overall, our findings demonstrate that calycosin promotes neuronal differentiation in PC12 cells, as evidenced by increased neurite outgrowth and transcriptional activity of neurofilament genes. Moreover, the involvement of TrkB signaling in calycosin‐induced neuronal differentiation highlights the potential therapeutic application of calycosin in neuroregeneration and neuronal disorders.

### 3.8 Calycosin Enhances the TrkB–MEK1/2–Erk1/2–CREB Signaling Pathway to Promote Neurogenesis in PC12 Cells and Exert Antidepressant Effects

**FIGURE 9:**
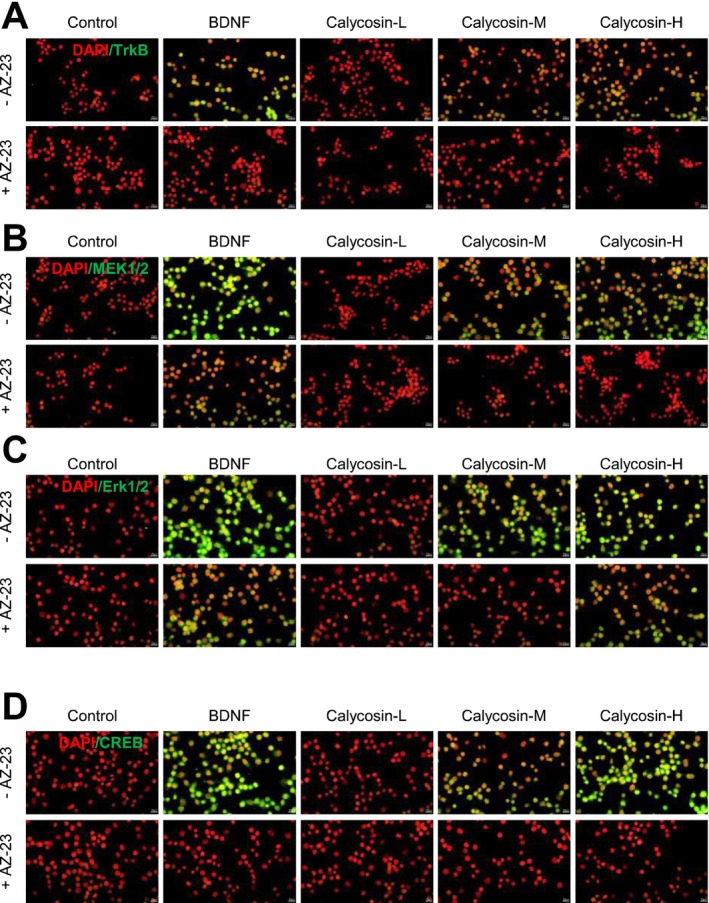
Calycosin increases in vitro TrkB–MEK1/2–Erk1/2–CREB translational levels. The cultures were pretreated with medium (control) or AZ‐23 (5 nM) for 3 h before adding BDNF (20 ng/mL) or calycosin (1, 3, 10 μM) for 48 h. The nuclei were stained with DAPI and shown in red. TrkB (A), MEK1/2 (B), Erk1/2 (C), and CREB (D) were revealed by the laser confocal microscope. Representative images were shown.

Neurogenesis is a fundamental element related to brain repair, and the BDNF‐associated signaling pathway is necessary for this process [47, 48]. BDNF and its downstream signaling, which includes TrkB, MEK1/2, Erk1/2, and CREB, influence several brain repair mechanisms, such as neurogenesis, synaptic plasticity, and synaptic transmission [20, 49]. These effects ultimately impact the progression of depression [5]. Therefore, we used a laser confocal microscope to investigate the effect of calycosin on the TrkB–MEK1/2–Erk1/2–CREB signaling pathway in cultured PC12 cells following a 48‐h treatment (Figure 9). As shown by the fluorescence signal (Figure 9), calycosin considerably increased the protein expression levels of TrkB (Figure 9A), MEK1/2 (Figure 9B), Erk1/2 (Figure 9C), and CREB (Figure 9D) in vitro in a dose‐dependent manner. AZ‐23 pretreatment significantly reduced the translational activity of the target genes induced by calycosin (Figure 9). As a positive control, BDNF administration increased the translational levels of CREB, MEK1/2, TrkB, and Erk1/2 in cultured PC12 after 48 h (Figure 9). However, the presence of AZ‐23 significantly decreased the translational levels of TrkB, MEK1/2, Erk1/2, and CREB in comparison to the untreated group (Figure 9).

**FIGURE 10:**
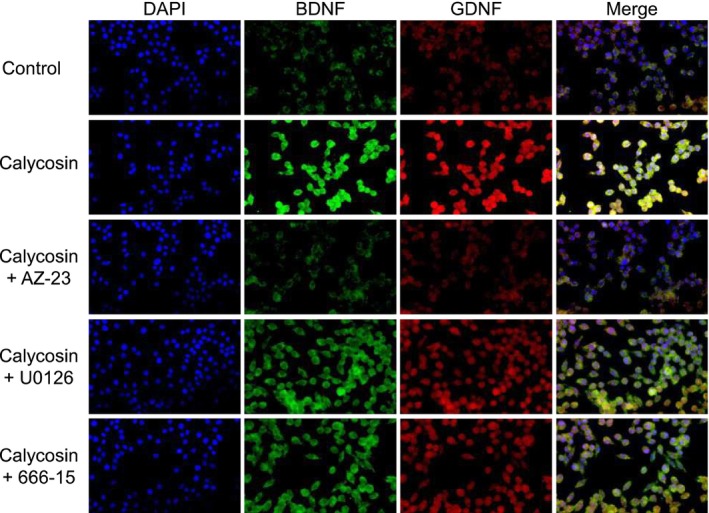
Application of specific inhibitors. The cells were pretreated with medium (control), AZ‐23 (5 nM), U0126 (5 μM), or 666‐15 (5 μM) for 3 h. Following pretreatment, calycosin (10 μM) was added and incubated for 48 h. Nuclei were stained with DAPI, and images were captured using a laser confocal microscope. Representative images are shown.

In addition to the previously conducted experiments, we have performed laser confocal microscopy to examine the protein expression levels of BDNF and GDNF following treatment with specific inhibitors targeting the TrkB–MEK1/2–Erk1/2–CREB signaling pathway (Figure 10). The purpose of these experiments was to gain a deeper understanding of the detailed signaling mechanisms involved. The results revealed that AZ‐23, a specific TrkB inhibitor, significantly reduced the protein levels of both BDNF and GDNF after 48 h of treatment, as compared to the calycosin group (Figure 10). This suggested that TrkB activation was a critical regulator of BDNF and GDNF expression in response to calycosin. On the other hand, the MEK‐Erk1/2 inhibitor U0126 was found to partially block the expression of both BDNF and GDNF (Figure 10). Similarly, 666‐15, a CREB inhibitor, was also able to partially inhibit BDNF and GDNF expression, further supporting the idea that MEK1/2‐Erk1/2‐CREB‐mediated signaling is involved in this process, but there may be additional regulatory mechanisms contributing to the observed functions (Figure 10). These findings highlighted the intricate and interconnected nature of the TrkB–MEK–Erk1/2–CREB signaling pathway in modulating BDNF and GDNF expression and suggested that while TrkB signaling played a dominant role, other pathways such as MEK‐Erk1/2 and CREB signaling also contribute to the regulation of these important neurotrophic factors. Taken together, the in vitro and in vivo data suggest that the TrkB–MEK1/2–Erk1/2–CREB signaling pathway may be involved in the anti‐depressive mechanisms of calycosin, correlating with its neurogenic bioactivity.

## 4 Discussion

The present study demonstrates the antidepressant‐like effects of calycosin in CMS animal models and highlights its potential as a novel therapeutic agent for depression. Isoflavones, such as calycosin, exhibited significant antidepressant‐like activities in CMS animal models, including the grooming frequency test, sucrose consumption test, tail suspension test, and open field test. These findings are consistent with previous studies indicating the antidepressant potential of isoflavones [50, 51, 52]. Clinical and epidemiological data also support the notion that isoflavones could be a well‐tolerated and effective option for managing depression [53]. The observed antidepressant‐like effects of calycosin, a type of isoflavone, suggest that it could serve as a promising alternative treatment for patients who do not respond favorably to conventional pharmacological interventions. A notable discovery of the study is the enhancement of neurogenesis associated with calycosin treatment, which further underscores its potential therapeutic benefits in the context of depression.

Neurogenesis has been identified as a key mechanism associated with the therapeutic effects of current antidepressants, while impaired neurogenesis has been implicated in the pathophysiology of depression [54, 55]. The results of the study suggest that the promotion of neurogenesis could be a key mechanism underlying the antidepressant effects of calycosin. The observed effects of calycosin on neurogenesis are consistent with previous studies indicating that calycosin stimulates neurogenesis through the activation of the BDNF signaling pathway [56]. Our study demonstrated that calycosin treatment enhances the TrkB–MEK1/2–Erk1/2–CREB signaling pathway, which may play a role in the anti‐depressant mechanisms of calycosin. BDNF is known to play a crucial role in neuronal survival, differentiation, and synaptic plasticity, and its involvement in the antidepressant effects of calycosin merits additional exploration [20]. The activation of these signaling pathways ultimately impacts the progression of depression [5, 48, 49, 57].

In our study, calycosin treatment considerably raised the protein expression levels of TrkB, MEK1/2, Erk1/2, and CREB in a dose‐dependent manner, as evidenced by the fluorescence signal. The translational activity of the target genes caused by calycosin was significantly reduced by AZ‐23 pretreatment, a specific TrkB inhibitor, indicating that calycosin exerts its effects through the TrkB receptor. Our results align with previous studies demonstrating that calycosin exerts its neurogenic effects through the activation of the BDNF signaling pathway [56]. Previous studies have also indicated that AZ‐23 exhibited Trk kinase inhibition and efficacy in mice after oral administration in a TrkA‐driven allograft model, as well as significant tumor growth inhibition in a Trk‐expressing xenograft model of neuroblastoma [58]. AZ‐23 is a potent and selective Trk kinase inhibitor from a novel series, showing promise for potential use as a cancer treatment [59, 60]. Another study provided strong evidence that AZ‐23 led to an increase in degenerating neurons, suggesting the involvement of BDNF in neuroprotection [61]. PC12 cells do not express full‐length TrkB receptors and require exogenous expression of TrkB to respond to BDNF [62]. This raises concerns about the direct activation of the TrkB signaling pathway by calycosin in the absence of endogenous TrkB receptors in PC12 cells. While the differentiation‐promoting effects of calycosin in PC12 cells have been demonstrated in this study, further investigations are warranted to elucidate the specific mechanisms by which calycosin mediates cellular differentiation in the absence of full‐length TrkB receptors. Furthermore, CREB, a transcription factor that plays a crucial role in synaptic plasticity and neurogenesis, has been implicated in the pathophysiology of depression and is considered a potential target for antidepressant therapies [5, 20, 49, 63, 64].

In addition to its antidepressant‐like effects, the study also demonstrated the hepatoprotective properties of calycosin, which could enhance its overall therapeutic potential in depression. Liver dysfunction has been associated with an increased susceptibility to depression, highlighting the importance of maintaining liver health for patients with depressive disorders [65, 66, 67]. The study demonstrated the hepatoprotective effects of calycosin through its ability to modulate liver enzymes and histomorphology in mice with liver injury. These findings are consistent with previous studies that have shown the hepatoprotective benefits of calycosin [24, 68]. Importantly, calycosin treatment was well‐tolerated by the experimental animals, with minimal adverse effects observed. This is a significant finding, as the side effects associated with current antidepressant medications often contribute to poor adherence and treatment discontinuation [69, 70].

Based on the findings, the results strongly suggest that calycosin holds promise as a novel therapeutic agent for depression, with various mechanisms of action including neurogenesis enhancement and hepatoprotection. These results are in agreement with previous studies that have reported the potential antidepressant and hepatoprotective effects of isoflavones [50, 51, 71, 72, 73]. Our study adds to the growing body of literature supporting the therapeutic potential of calycosin and underscores the importance of exploring natural products as alternative treatment options for depression. Overall, our study provides evidence for the antidepressant‐like effects of calycosin in CMS animal models and highlights its potential as a novel therapeutic agent for depression. The enhancement of neurogenesis, as well as its hepatoprotective effects, may contribute to the entire therapeutic potential. Calycosin's underlying anti‐depressive effects may include the TrkB–MEK1/2–Erk1/2–CREB signaling pathway. Our findings indicate the necessity for additional research into calycosin's particular molecular targets, as well as clinical efficacy and safety analyses.

## 5 Conclusion

In summary, depression presents a significant challenge to treat due to limited therapeutic options and various negative impacts. This study highlights the antidepressant‐like properties of calycosin, a bioactive compound derived from *Astragalus membranaceus*, in an animal model of depression. Calycosin treatment effectively alleviated depressive symptoms, promoted neurogenesis, and exhibited hepatoprotective effects in mice with liver injury. The antidepressant effects of calycosin were linked to the activation of the BDNF signaling pathway and the upregulation of the TrkB–MEK1/2–Erk1/2–CREB pathway. These findings suggest that calycosin holds promise as a novel therapeutic agent for depression, acting through multiple pathways including hepatoprotection and neurogenesis enhancement. Further research is necessary to elucidate the specific molecular targets of calycosin, assess its efficacy and safety in clinical settings, and determine the optimal dosage and administration routes for potential clinical use. Ultimately, these findings may lead to the development of new, effective, and well‐tolerated treatments for depression, addressing the unmet needs of individuals who do not respond adequately to current pharmacological therapies.

## Author Contributions

**Guowei Gong:** conceptualization, data curation, investigation, writing – original draft. **Yaqun Liu:** formal analysis. **Zhenxia Zhang:** writing – review and editing. **Yuzhong Zheng:** conceptualization, writing – review and editing.

## Ethics Statement

All experiments were approved by the Guidelines for Laboratory Animal Care and Committee on the Use of Live Animals in Teaching and Research.

## Conflicts of Interest

The authors declare no conflicts of interest.

## Supporting information


**Figure S1.** Cell viability determination. Calycosin (0–30 μM) was applied to cells for a duration of 48 h. The data are displayed in mean ± SD, *n* = 3, and as a percentage change from the control group. A significant result was ***p < 0.001 when compared to the control group.

## Data Availability

The data used and/or analyzed during the current study are available from the corresponding author upon reasonable request.
