# Supplementary figures and images for: Therapeutic Targets and Molecular Mechanisms of Calycosin in the Treatment of Depression: Insights From Chronic Mild Stress Animal Models
Source: CNS Neurosci Ther. 2025 Apr 22;31(4):e70353. doi: 10.1111/cns.70353 (PMC12012568; doi:10.1111/cns.70353)

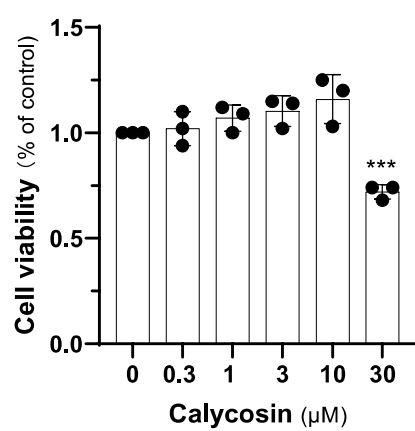

Supplement: Supplementary file 1 — Figure S1. Cell viability determination. Calycosin (0–30 μM) was applied to cells for a duration of 48 h. The data are displayed in mean ± SD, n = 3, and as a percentage change from the control group. A significant result was ***p < 0.001 when compared to the control group. [file CNS-31-e70353-s001.pdf]
